# Measuring patient engagement: development and psychometric properties of the Patient Health Engagement (PHE) Scale

**DOI:** 10.3389/fpsyg.2015.00274

**Published:** 2015-03-27

**Authors:** Guendalina Graffigna, Serena Barello, Andrea Bonanomi, Edoardo Lozza

**Affiliations:** ^1^Department of Psychology, Università Cattolica del Sacro CuoreMilan, Italy; ^2^Department of Statistical Sciences, Università Cattolica del Sacro CuoreMilan, Italy

**Keywords:** patient engagement, patient activation, patient engagement measurement, patient health engagement scale, psychometric properties, ordinal scale

## Abstract

Beyond the rhetorical call for increasing patients' engagement, policy makers recognize the urgency to have an evidence-based measure of patients' engagement and capture its effect when planning and implementing initiatives aimed at sustaining the engagement of consumers in their health. In this paper, authors describe the Patient Health Engagement Scale (PHE-scale), a measure of patient engagement that is grounded in rigorous conceptualization and appropriate psychometric methods. The scale was developed based on our previous conceptualization of patient engagement (the PHE-model). In particular, the items of the PHE-scale were developed based on the findings from the literature review and from interviews with chronic patients. Initial psychometric analysis was performed to pilot test a preliminary version of the items. The items were then refined and administered to a national sample of chronic patients (*N* = 382) to assess the measure's psychometric performance. A final phase of test-retest reliability was performed. The analysis showed that the PHE Scale has good psychometric properties with good correlation with concurrent measures and solid reliability. Having a valid and reliable measure to assess patient engagement is the first step in understanding patient engagement and its role in health care quality, outcomes, and cost containment. The PHE Scale shows a promising clinical relevance, indicating that it can be used to tailor intervention and assess changes after patient engagement interventions.

## Introduction

In the last decades, the increased epidemiology of chronic conditions, due to the aging of the population and to the diffusion of environmental stressors, has implied an enhanced organizational and economic effort of the healthcare system (Lassman et al., 2014; Pallin et al., 2014). This effort implies a burden difficult to be dealt, also due to the general reduction of resources that the healthcare organizations have to face in the present period of economic crisis. In other words, healthcare organizations today have to “do more with less” (Steinmann et al., 2007; Hartholt et al., 2011). In this scenario, healthcare experts, managers, and policy makers are recognizing the importance of a paradigm shift in the planning and delivery of healthcare in the favor of promoting a more active role of patients in the management of their healthcare (Barello et al., 2014a; Menichetti et al., 2014). In the field of medicine and public health management, recent theorizations have indeed advocated considering patients as important human resources that should be actively involved in the healthcare organization and along the process of care delivery (Crawford et al., 2002; Davis et al., 2005; Clancy, 2011; Bellardita et al., 2012; Graffigna et al., 2013a; Barello et al., 2014b, 2015a). There is a shared agreement that making patients better informed and more directly responsible for their health and care management is pivotal to make healthcare organizations better sustainable at the economic, organizational, and psychological level (Coulter et al., 2012; Graffigna et al., 2014). To engage patients in healthcare is considered across the world as a key strategy to improve patients' adherence, clinical outcomes, and satisfactions toward the received care. (Renedo and Marston, 2011; Coulter, 2012a,b; Ocloo and Fulop, 2012; Barello and Graffigna, 2014). Furthermore, the achievement of a concrete engagement of patients in their healthcare management has also been envisaged as an effective strategy to reduce healthcare costs (Burns et al., 2014; Provenzi et al., 2015).

However, current practices devoted to improve patient engagement in their healthcare management show a lack of shared guidelines to achieve this goal (Hor et al., 2013; Hardyman et al., 2015). Furthermore, experts testify a certain confusion about what patient engagement is and how it may be conceptualized and achieved, as testified by the plethora of terms often used as interchangeable in this domain (Graffigna et al., 2013a, 2015; Menichetti et al., 2014). Finally, only few empirical studies aimed to measure healthcare performances and their ability to improve the engagement of patients, with results that are poorly comparable and generalizable (Staniszewska et al., 2008). This is also due to the lack of instruments able to assess the level of patient engagement in healthcare management. Particularly, this lack of concrete instruments to assess patient engagement is potentially detrimental to the broadly accepted assumption that health care practices need to be align with evidence-based insights about individuals' healthcare preferences and needs.

**Figure 1:**
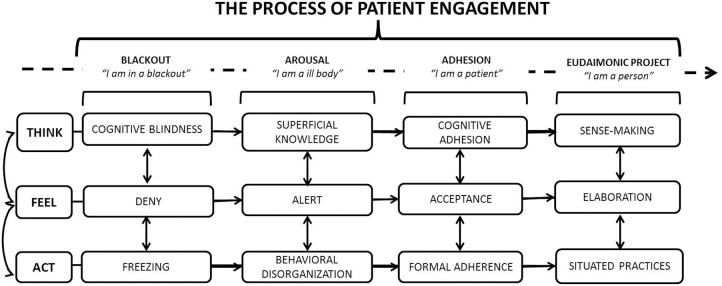
**The patient health engagement model, adapted from Graffigna et al. (2013b)**.

So far, in the international scenario, only one assessment instrument in this conceptual area exists, the Patient Activation Measure (PAM). The PAM, developed by Hibbard et al. (2004, 2005), is a powerful instrument able to detect the level of activation of patients toward their care management (Hibbard and Mahoney, 2010; Greene and Hibbard, 2012). This scale has been widely used in the US to orient medical practices, and it has now been validated in several countries (Maindal et al., 2009; Brenk-Franz et al., 2013; Ahn et al., 2014; Magnezi and Glasser, 2014; Graffigna et al., under review). However, although the concepts of “activation” and “engagement” have some areas of conceptual overlapping, they differ according to the breath of the healthcare relation considered. The concept of “activation” is mainly limited to the prototypical situation of a doctor-patient consultation while the concept of “engagement” seeks to consider multiple levels of the patients' fruition of the healthcare (Menichetti et al., 2014). Furthermore, the concept of activation is related mainly to the cognitive and behavioral components of patients' attitude toward healthcare, and it is mainly conceptualized as an incremental attitude that the patient may develop. On the contrary, the concept of engagement offers a more holistic consideration of the psychological elaboration of the patient about his/her health condition and presents a multi-stage development (Graffigna et al., 2014). Precisely, patient engagement is a “process-like and multi-dimensional experience, resulting from the conjoint cognitive (think), emotional (feel), and conative (act) enactment of individuals toward their health management. In this process, patients go through four subsequent positions (i.e., blackout, arousal, adhesion, and eudaimonic project; see Figure 1). The unachieved synergy among the different subjective dimensions (think, feel, act) at each stage of the process may inhibit patients' ability to engage in their care” (Graffigna et al., 2014, p. 1). Precisely, in previous studies, we developed the Patient Health Engagement (PHE) model based on evidence about patients' experiences and preferences regarding their engagement in care management, which may be considered as a compass to help healthcare practitioners and policy makers customize their interventions to engage patients in care management (Graffigna et al., 2014). According to the PHE model's process view of patient engagement, individuals may be differentially engaged in care management according to their emotional, cognitive, and behavioral mindset. For instance, when a patient receives a serious diagnosis, s/he might not be able to engage fully in care management because of destabilizing emotional effect on health knowledge (blackout position). The healthcare system at this stage needs to provide a more systematic assistance and scaffolding that would include the caregivers' main needs and priorities. Across the patient's engagement journey, on the contrary, as patients gain knowledge, they generally become more emotionally stabilized and thus more confident in their ability to engage in managing their disease condition (i.e., positions of arousal, adhesion, eudaimonic project).

According to the PHE model, we argue that practitioners and healthcare services that are better attuned to the engagement stage of patients may develop a more effective flexibility in their strategies to promote the active role of patients in their care management. Clearly, there is no monolithic engagement trajectory for all patients. However, an important precursor to improving healthcare is to undertake an engagement-sensitive approach based on the patients' desires and expectations. Failure to recognize and support the patients' engagement status might result in misalignment patient-provider expectations, dissatisfaction with care, and poor adherence to treatments, which undermine the real translation of the patient engagement goal into practice.

Based on this framework, patients' activation may be considered a precursor of patients' engagement, but it does not coincide with the phenomenon of engagement (Graffigna et al., 2015; Menichetti et al., 2014). Furthermore, due to the complex and dynamic psychological nature of the patient engagement experience, specific assessment tools are needed that are able to grasp the multifaceted nature of the emotional and motivational experience of patients during their healthcare management journey.

Based on these premises, the present paper describes the psychometric proprieties of a new scale developed based on the PHE model (the PHE-scale). In particular, the aim of the present study is three-fold: (1) to evaluate the psychometric properties of the PHE-scale and (2) to evaluate the association between PHE-scale scores and concurrent measures.

## Materials and methods

### Development of the PHE-scale

The items and the structure of the scale were developed based on a systematic analysis of the literature (Barello et al., 2014a,b) and an extensive qualitative study aimed at deeply exploring chronic patients' journey about their care management (Barello et al., 2014b; Graffigna et al., 2014, under review). The scale thus presents a bottom up development and is particularly able to grasp inner psychological experiences of patients along their engagement stages.

The scale adopted an ordinal structure in order to be consistent with the PHE model's conceptualization, which envisages four different positions along the engagement continuum. Items were formulated based on the interviewees' spontaneous narratives in order to describe the subjective positions that a patient may experience along his/her engagement journey. Although the PHE model described four engagement positions, the ordinal scale was measured on a 7-point scale (see Supplementary Material) in order to facilitate patients' responses and to avoid social desirability bias (Furnham, 1986). For example, to declare a low position of engagement (e.g., the blackout) might be considered poorly socially acceptable by respondents, and it might thus be avoided. On the contrary, the possibility to rate one-self in an intermediate position (e.g., between the blackout and the arousal) may facilitate an accurate self-description. For these reasons, in the following analyses, we coded intermediate positions between the four stages as equivalent to the previous engagement position, i.e., a score of 2 on the 7 points scale means that respondent positions him/herself in the first stage of engagement while a rate 6 means a position in the third stage.

The PHE-scale originally consisted of 9 ordinal items, and it was reduced to 5 ordinal items after the first pilot phase (see following paragraphs). The 5 final items are presented in the Supplementary Material, both in the original (Italian) formulation and in their English translation. The PHE-scale was included in a longer questionnaire that included concurrent measures (PAM-13 and MMAS-4) and *ad hoc* items related to socio-demographics and clinical descriptive variables.

### Concurrent measures

#### Patient activation measure

Developed by Hibbard et al. (2005), the 13-item Patient Activation Measure (PAM) is an interval-level, unidimensional Guttman-like measure that contains items measuring self-assessed knowledge about chronic conditions, beliefs about illness and medical care, and self-efficacy for self-care. The PAM focused on physical conditions, and it was designed to measure activation as a broad construct. In the present study, we used the Italian validated version of the PAM (Graffigna et al., under review).

#### Morisky medication adherence scale

Medication-taking behavior was assessed using the 4-item Morisky Medication Adherence Scale (MMAS-4) (Morisky et al., 1986, 2008; Shalansky et al., 2004). This simple 4-question survey assesses the likelihood of patients taking their drug therapy as prescribed. The items measure the degree to which patients self-report non-adherence with prescribed medication due to forgetting, carelessness, stopping the drug when feeling better or stopping the drug when feeling worse. In the present study, we used the Italian validated version of the MMAS-4 (Fabbrini et al., 2013).

### Demographic and clinical variables

A set of *ad hoc* items were included in the questionnaire in order to describe socio-demographic and clinical characteristics of the patients. Those also served as screening variables in order to select panel respondents. These items were related particularly to the following patients' characteristics: age; gender; education; marital status; type of diagnosis; year from the first diagnosis.

### Procedure

The data were collected from a panel of chronic patients. To be included in the panel, patients had to be: (1) Italian and reside in Italy; (2) diagnosed with one or more chronic diseases; (3) not diagnosed with a major psychiatric disturbance; (4) following a chronic treatment for their disease/s; (5) aged >18 years old; and (6) of both genders.

The data collection was performed in three phases through the QUALTRICS online system. The first pilot phase was conducted on a sample of 48 chronic patients using the long version of the PHE-scale (9 ordinal items) together with the concurrent measures and the *ad hoc* items related to socio-demographic and clinical variables. After the completion of the questionnaire, in the pilot phase, patients were also required to discuss the readability of the instrument and to indicate potential problems with answering the scale items. This preliminary assessment allowed us to select 5 items. A new data collection wave based on the revised version of the questionnaire was then conducted on a sample of 352 chronic patients. In addition, the final version of the PHE-scale underwent a final test-re-test data collection phase on a sub-sample of 30 chronic patients.

### Ethical concerns

The study received approval from the Università Cattolica del Sacro Cuore Ethics Committee. Patients consented to participate in the study, and they were allowed to withdraw from the study whenever they wanted. The data were collected anonymously and analyzed in an aggregated way.

## Results

### Participants

Overall, 510 patients were invited to participate in the study but only 430 met the inclusion criteria and completely answered the questionnaire for the psychometric analysis. Demographic and clinical characteristics are summarized in Table 1.

**Table 1:** **Demographic and clinical characteristics of the sample**.

**Demographic variables**	
Mean age (years)	51.3
Gender (% female)	46.6
**MARITAL STATUS (%)**	
Never married	21.1
Married	68.6
Divorced	8.1
Widowed	2.2
**EMPLOYMENT (%)**	
Employed	43.6
Retired	33
Homemaker	8.3
Student	5.6
Unemployed	6.6
Other	2.9
**EDUCATION (%)**	
None	0.9
Primary school	6
Middle school	12.1
High school	48.4
Graduate or higher	32.6
**Clinical variables**	
**DISEASE (%)**	
Asthma	25.5
Celiac disease	4.8
Hypertension	35.6
COPD	8.1
Type I diabetes	3.7
Type II diabetes	24.2
Cardiovascular disorder	15.3
Cancer	9.6
Chron disease	2.9
Fibromyalgia	7.6
Ulcerous colitis	4.5
Lupus	2.2
Osteoarthritis	10.8
Rheumatoid arthritis	11.1
Myeloid chronic leukemia	0.6
Hypercholesterolemia	22.1
Hepatitis	3.4
Anaemia	9.3

### Pilot study

A preliminary pilot study was conducted in order to calibrate the PHE scale and to eliminate unnecessary items. Moreover, upon the completion of the questionnaire, respondents were also required to discuss the readability of the scale items and to indicate potential problems in answering. The aim of this first study was to obtain an ordinal scale comprising a low number of items measuring the latent construct of interest. The initial scale comprised 9 ordinal items (Table 2).

**Table 2:** **The 9-item version of the PHE scale**.

**9-item scale**	**5-item scale**	**Items**						
**WHEN I THINK ABOUT MY DISEASE**								
A	1	I feel in blackout		I am in alarm		I am aware		I feel positive
		O	O	O	O	O	O	O
B	2	I feel dazed		I am in trouble		I am conscious		I feel serene
		O	O	O	O	O	O	O
C	–	I can't understand what happened to me		I can't manage the information that my physician gives me		The information my physician gives me is clear to me		Despite my illness, I know how to manage my life
		O	O	O	O	O	O	O
D	–	I feel totally messed up		I am not always able to use the information my physician gives me		I understand what my physician tells me to do		I understood how to manage my life despite my illness
		O	O	O	O	O	O	O
E	–	I feel totally in a maze		I find it hard to gather up the information my physician gives me		It is clear to me what my physician tells me to do		I know everything I should do to best manage my life despite my illness
		O	O	O	O	O	O	O
F	–	I let others take care of me	O	I try to manage my disease but I feel that I am not totally able		I strictly follow the rules that my physician gives me		I can autonomously manage my medical regimen
		O	O	O	O	O	O	O
G	3	When I think about my illness I feel overwhelmed by emotions		I feel anxious every time a new symptom arises		I got used to my illness condition		Despite my illness I perceive coherence and continuity in my life
		O	O	O	O	O	O	O
H	4	I am very discouraged due to my illness		I feel anxious when I try to manage my illness		I feel I adjusted to my illness		I am generally optimist about my future and my health condition
		O	O	O	O	O	O	O
I	5	I feel totally oppressed by my illness		I am upset when a new symptom arises		I feel I have accepted my illness		I can give sense to my life despite my illness condition
		O	O	O	O	O	O	O

The sample of the pilot study comprised 48 subjects (65% males; 35% females, aged from 21 to 87 years old; *M* = 58.8 years, *SD* = 21.1). Since the 9 proposed items had an ordinal nature, the data analysis involved suitable technique for ordinal data. In particular, the calibration of the scales and the exploration of the factorial structure were carried out using a Categorical Principal Component Analysis (CATPCA) and the reliability analysis was conducted using the Ordinal Alpha via Empirical Copula Index (Bonanomi et al., 2012, 2014). The latter is a reliability index for polytomous ordinal items based on the Spearman grade correlation coefficient, and it considers copula-based measures of association across the ordinal variables. This approach relaxes several restrictive hypotheses that are present both in the case of classical Cronbach's Alpha (for metric data) and in the case of Ordinal Alpha proposed by Zumbo et al. (2007). In this study, an empirical version of the index, the Ordinal Alpha via Empirical copula, was evaluated; this version avoids the researcher to make assumptions about the type of dependence relating the latent variables underlying the ordinal indicators.

Table 3 shows descriptive statistics of all items included in pilot survey, as well as their Shannon Entropy index. Items with ceiling effects (medians = 4) were excluded from further analyses (D–F). Moreover, item C was excluded due to a lower Shannon Entropy index and because its elimination increased the reliability of the scale via an Ordinal Alpha analysis. The final version of the PHE scale comprised 5 items with promising psychometric properties (considering the sample size and the explorative nature of the pilot study): ordinal alpha of 0.82 and CATPA (Table 4) suggested a monodimensional latent structure, with eigenvalue of 3.69 and 73.84% of explained variance.

**Table 3:** **Item-level descriptive statistics for ranks on the PHE 9-item scale**.

**PHE item**	**Rank range**	**Minimum**	**Maximum**	**Median**	**Shannon entropy**
Item A (1)	1–4	1	4	3	0.81
Item B (2)	1–4	1	4	3	0.81
Item C	1–4	1	4	3	0.77
Item D	1–4	1	4	4	0.76
Item E	1–4	2	4	4	0.74
Item F	1–4	2	4	4	0.74
Item G (3)	1–4	1	4	3	0.90
Item H (4)	1–4	1	4	3	0.90
Item I (5)	1–4	1	4	3	0.95

**Table 4:** **Factor loadings from CATPA—one factor solution—pilot study**.

**PHE item**	**One factor solution**
Item A (1)	0.85
Item B (2)	0.76
Item G (3)	0.91
Item H (4)	0.92
Item I (5)	0.84

In the pilot phase, when requested to assess the questionnaire, patients declared that the items were understandable and contained familiar wording and descriptions of the patient engagement that were well attuned to their sentiment.

### Validation study

The validation study was conducted on the final 5-item version of the scale (see Supplementary Material).

To test and verify the unidimensionality of the scale, three analyses were conducted: (a) an exploratory CATPCA, (b) a confirmatory CFA for ordinal data, and (c) a Rasch Model.

Furthermore, we assessed the internal consistency and test-retest reliability.

The validation study involved 382 participants with chronic disease. The sample was divided into three major subgroups: Group 1 (*n* = 206) was used to conduct the exploratory analysis, Group 2 (*n* = 146) was used to conduct the confirmatory analysis. The third group involved 30 subjects to examine test-retest reliability.

#### Exploratory categorical principal component analysis

Descriptive statistics of the individual items were calculated to conduct the initial exploration of the data. Table 5 provides the item-level descriptive statistics for all items. Since the ordinal nature of the items, the median and the Shannon Entropy Index were calculated.

**Table 5:** **Item-level descriptive statistics for ranks on the PHE**.

**PHE item**	**Rank**	**Minimum**	**Maximum**	**Median range**	**Shannon entropy**
Item 1	1–4	1	4	3	0.79
Item 2	1–4	1	4	3	0.85
Item 3	1–4	1	4	3	0.85
Item 4	1–4	1	4	3	0.92
Item 5	1–4	1	4	3	0.88

Table 6 provides the inter-item polychoric correlation matrix. The polychoric correlation (Pearson, 1900) is a measure of bivariate association arising when both observed variables are ordered categorical variables derived from polychotomizing latent underlying continuous variables.

**Table 6:** **Item-item polychoric correlation matrix for ranks on the PHE**.

**PHE item**	**Item 1**	**Item 2**	**Item 3**	**Item 4**	**Item 5**
Item 1	–	0.61	0.66	0.71	0.67
Item 2		–	0.54	0.60	0.61
Item 3			–	0.78	0.80
Item 4				–	0.78
Item 5					–

The average inter-item polychoric correlation is a subtype of internal consistency reliability. It is obtained by taking all of the items on a test that probes the same construct, determining the polychoric correlation coefficient for each pair of items and finally taking the average of all of these polychoric correlation coefficients. Every polychoric correlation coefficient was higher than 0.5. The average inter-item polychoric correlation is equal to 0.68, which indicates a high correlation between items.

An exploratory categorical principal component analysis (CATPCA) was conducted on the final 5-item version of the PHE scale on Group 1 (*n* = 206, 54% males and 46% females aged 21–84 years old; *M* = 52.5 years, *SD* = 14.9). A CATPCA was chosen because of the ordinal nature of the items. An initial analysis was performed without any restriction on the number of metric factors to be estimated. The initial analysis yielded one factor with eigenvalue 3.37, which is over Kaiser Criterion of 1, explaining 67.4% of the total variability. The scree plot confirmed the one factor structure. Table 7 shows the factor loadings for the one solution of the CATPCA.

**Table 7:** **Factor loadings from CATPCA—one factor solution**.

**PHE item**	**One factor solution**
Item 1	0.74
Item 2	0.71
Item 3	0.84
Item 4	0.89
Item 5	0.88

*All factor loadings had a very high value (>0.7), confirming the unidimensionality of the scale*.

#### Confirmatory factorial analysis

**Figure 2:**
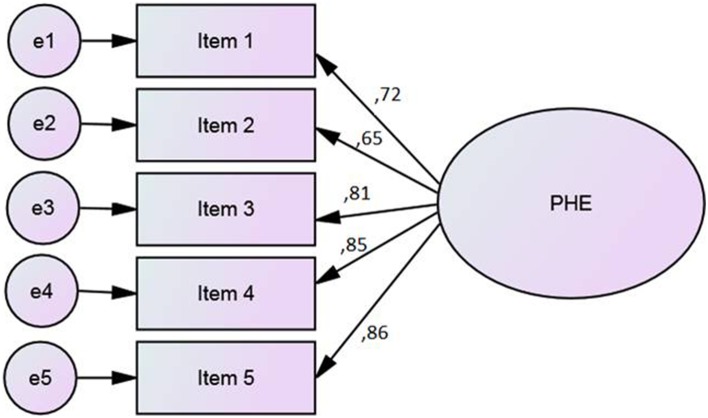
**CFA on PHE scale: Standardized estimates**.

A Confirmatory Factor Analysis (CFA) (Figure 2) was performed on Group 2 (*n* = 146, 54% males; 46% females, aged from 21 to 84 years old; *M* = 51.3 years, *SD* = 16.6) to study the replicability of the factor structure obtained by CATPCA. The estimation method was asymptotically distribution free, particularly suitable for ordinal data and not-Gaussian distributions. To evaluate the closeness of the hypothetical model to the empirical data, multiple goodness-of-fit indexes were used, including the ratio of the chi-square to degrees of freedom (χ^2^/df), the Comparative Fit Index (CFI), the Standardized Root Mean Square Residual (SRMR), and the Root Mean Square Error of Approximation (RMSEA). To test the model, each variable was allowed to load on only one factor, and one variable loading in the latent factor was fixed at 1.0. For the remaining factor loadings, residual variances were freely estimated.

CFA showed reasonable goodness of fit indices. The fit indices met the criteria of fit for the hypothesized one-factor structure. Chi square (χ^2^= 10.98, *df* = 5, *p* = 0.052) value and goodness of fit indices (CFI = 0.981, RMR = 0.018, RMSEA = 0.059) suggested that the model is coherent with the data. The analysis of modification indices did not find the relation between the error covariance of the items.

To verify the validity and generalizability of the factor structure, a multigroup confirmatory analysis tested measurement invariance in the two subsamples divided by gender. Table 8 shows the verified invariance hypothesis. The Δχ^2^ between the unconstrained and constrained models did not yield significant results. The factor structure was invariant by gender.

**Table 8:** **Multigroup CFA by gender**.

**Model**	**χ ^2^**	***df***	**RMSEA**	**CFI**	**Δχ^2^(df)**	***p***
Unconstrained	10.9	5	0.059	0.971	–	–
Invariant factor loading	27.1	15	0.049	0.945	16.2 (10)	0.09

#### Rasch model

A Rasch Model was implemented to further investigate whether the PHE scale was uni-dimensional, and whether all items fit the model well.

Two (infit and outfit) mean square (MNSQ) statistics were computed to check whether the items fit the expected model. MNSQ determines how well each item contributes to defining a single underlying construct (unidimensionality). Infit is more sensitive to misfitting responses to items closest to the person's ability level while outfit is more sensitive to misfitting items that are farther away. If the data fitted the Rasch Model, the fit statistics should be between 0.6 and 1.4. According to Wright et al. (1994), regarding clinical observations, the fit statistics could be between 0.5 and 1.5. Table 9 shows the results of the Rasch Analysis. The measure of each item represents the estimate for the item difficulty expressed in logits; SEM is the standard error of measurement in estimation of the item difficulty; Infit and Outfit are measures of item fit.

**Table 9:** **PHE scale—Rasch Analysis**.

**PHE item**	**Measure (logits)**	**SEM**	**Infit MNSQ**	**Outfit MNSQ**
Item 1	1.88	0.11	0.90	0.89
Item 2	0.55	0.10	1.14	1.14
Item 3	0.68	0.10	0.74	0.72
Item 4	1.48	0.10	0.63	0.63
Item 5	0.93	0.10	0.62	0.65

Infit and Outfit statistics ranged from 0.62 to 1.14, which all are within the acceptable range. The person separation index (PSI) was calculated to evaluate the reliability in the Rasch Model (PSI = 0.884).

Rasch Model confirmed the unidimensionality of PHE scale and the fit of each item of the scale to the data.

#### Internal consistency and test-retest reliability analysis

As in the pilot study, PHE scale had a very good internal consistency, since the value of the Ordinal Alpha via Empirical Copula was equal to 0.85. In Table 10, the Ordinal Alpha was evaluated after deleting individual items. Each item contributed significantly to the PHE scale score. The internal consistency of the 5-item PHE scale was satisfactory.

**Table 10:** **Ordinal Alpha via Empirical Copula if item deleted**.

**Item**	**Ordinal Alpha if item deleted**
Item 1	0.82
Item 2	0.82
Item 3	0.78
Item 4	0.77
Item 5	0.77

Test-Retest reliability was examined by calculating two-way mixed, absolute concordance intra-class correlation coefficient (ICC). A sample of 30 participants was retested after 15 days. This subsample did not differ significantly from the initial sample in terms of gender [χ ^2(1)^ = 0.004, *p* = 0.94]. According to Fleiss parameters (1986), ICC yielded excellent results after 15 days (ICC = 0.95; CI = 0.90−0.97).

#### Concurrent validity

To assess concurrent validity, PHE factor scores were evaluated in relation to PAM and MMAS-4 scores.

First, a Pearson correlation was calculated. The results showed a moderate correlation between Patient Engagement and Patient Activation Measures (*r* = 0.431, *p* < 0.001). In other words, higher levels of Engagement were moderately and significantly related to higher levels of Activation.

For Patient Activation Measures, an independent samples *t*-test was conducted to measure the PHE factor scores for patients with medium or high adherence to medication (MMAS-4 score =0) and patients with low adherence (MMAS-4 score >0). This classification is considered clinically relevant (Spatola et al., 2014). Patients with low MMAS-4 scores, i.e., indicating good adherence to medication, scored significantly higher on PHE (*M* = 0.16, *SD* = 1.01) compared to patients with low adherence [*M* = −0.10, *SD* = 0.99; *t*_(350)_ = 2.299, *p* = 0.022]. Furthermore, the correlation between PHE and MMAS-4 scores was significant (*r* = −0.165, *p* < 0.01) and negative, in accordance with theoretical expectations.

## Discussion

Although patient engagement has been considered a key factor to improve healthcare delivery, currently there is a lack of instruments able to assess patient engagement. To date, only one instrument, the Patient Activation Measure (PAM), has been developed by Hibbard et al. (2004, 2005) to measure the active role of patients in their care. This instrument, although extremely valuable and widespread in the clinical practice in several countries (Maindal et al., 2009; Zill et al., 2013; Ahn et al., 2014; Magnezi and Glasser, 2014), does not appear able to grasp the complexity and dynamicity of the psychological experience of patient engagement. Particularly patient activation, which is the concept underlying the PAM development, mainly relates to the behavioral and cognitive attitude of a patient in his/her care management. From previous studies aimed at unveiling the inner subjective experience of engagement of chronic patients (Barello et al., 2014a, 2015a,b; Graffigna et al., 2014) we discovered that the emotional elaboration of the disease diagnosis and of its psycho-social effect on patients' life, plays a fundamental role in the development of patients' engagement. Thus, from our perspective, the path of motivational and emotional elaboration of the new patient's identity occurring after the disease diagnosis (and of the consequent reframing of daily routines, values, and projects) has to be considered in order to understand patients' engagement (Barello and Graffigna, 2014).

Based on the Patient Health Engagement (PHE) model that we theorized previously, we developed a specific assessment tool (Graffigna et al., 2013c). Particularly, the PHE scale was structured based on the systematic analysis of the literature that has focused on patient engagement and the collection of spontaneous patient narratives. Particularly, the set of qualitative researches previously conducted to explore the chronic patients' engagement journey helped us generate items well attuned to patients' feelings and to the way in which they express them.

The psychometric analysis of the PHE scale conducted in this study aimed to (1) evaluate the psychometric properties of the PHE-scale and (2) evaluate the association between PHE-scale scores and concurrent measures.

Based on the pilot analysis, 5 items that presented promising psychometric properties were included in the final version of the PHE scale. To answer the first objective of the study, three analyses were conducted: (a) an exploratory CATPCA, (b) a confirmatory CFA for ordinal data, and (c) a Rasch Model. Furthermore, we assessed the internal consistency and test-retest reliability of the scale.

The exploratory categorical principal component analysis (CATPCA) yielded one factor, which was confirmed in the subsequent CFA conducted on an independent sample, thus suggesting the unidimensionality of the scale and good fit of the model with the data. Moreover, the Rasch Model confirmed the unidimensionality of PHE scale, and the importance in terms of fitting of each item of the scale. Finally, a good internal consistency of the PHE scale was found, as indicated by satisfactory Ordinal Alpha and the test-retest analysis.

Finally, to assess the concurrent validity of the PHE scale, PHE factor scores were evaluated in relation to PAM and MMAS-4 scores. A moderate correlation was found between Patient Engagement and Patient Activation Measures, thus confirming our theoretical assumption that patient engagement, although with some degree of conceptual overlapping, consists in a different and more complex psychological phenomenon compared to the patient activation. This result seems also be related to the process-like nature of patient engagement that underlies the development of the PHE scale, a process that differs from the mere incremental nature of patient activation.

Moreover, the correlation between PHE and MMAS-4 scores was significant and negative (which is consistent with theoretical expectations), although the correlation coefficient was small. This evidence confirms the theoretical assumption that patient engagement is related to the adherence of patients in treatment management, although it shows that the experience of patient engagement overcomes the singular setting of treatment management and relates to a wider kind of relation (“exchange”) between an individual and the healthcare system during his/her healthcare journey (Graffigna et al., under review).

The current study is the first to evaluate the psychometrics properties of the PHE scale. Further analyses are needed to explore the strength of these evidences on other cohorts of patients and in other countries. However, these findings appear promising and suggest a high clinical relevance of the instrument. Particularly, thanks to the bottom up developmental process, the PHE scale results are able to grasp the complex psychological experience of the patient engagement journey, as emerged from the pilot phase of the study. Furthermore, thanks to its shortness, the scale can be easily used in the practice of the clinical encounter in order to train healthcare professional in patient-centered communication strategies (Lamiani et al., 2012) aimed at enhancing patients' engagement in self-management. Finally, the PHE scale is also featured by methodological innovativeness thanks to its ordinal structure, that results well coherent with the PHE model conceptualization. In addition patients indicated that it was particularly easy to answer to the questions of the scale, which described well the different psychological positions that the individuals may experience during the healthcare journey. We further recommend that our scale be validated among other clinical populations and healthcare settings. Moreover, future studies should research on the relationship between patient engagement and other patients' variables—such as health-related locus of control, coping styles, adherence to treatments—in order to identify antecedents and outcomes of patient engagement.

### Conflict of interest statement

The authors declare that the research was conducted in the absence of any commercial or financial relationships that could be construed as a potential conflict of interest.
