# Supplementary material for: Measuring patient engagement: development and psychometric properties of the Patient Health Engagement (PHE) Scale
Source: Front Psychol. 2015 Mar 27;6:274. doi: 10.3389/fpsyg.2015.00274 (PMC4376060; doi:10.3389/fpsyg.2015.00274)
Supplement: Supplementary file 1 [file DataSheet1.DOCX]

**Appendix**

**Patient Health Engagement Scale (PHE-Scale) – the original Italian version**

Qui di seguito troverà 5 affermazioni che descrivono come una persona può sentirsi quando pensa alla sua salute. Ciascuna frase può essere completata scegliendo uno dei 4 stati specifici, oppure i punti intermedi fra i diversi stati. Le chiediamo di indicare la Sua posizione rispetto allo stato che più La rispecchia, indicando il pallino corrispondente.


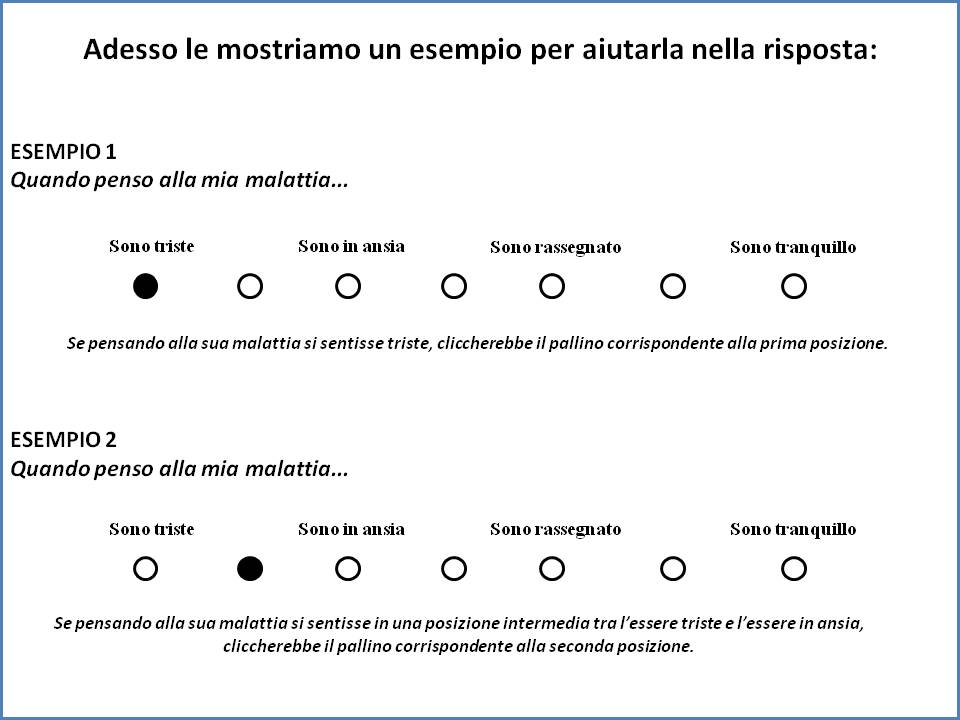


|  | | | | | | | | |
| --- | --- | --- | --- | --- | --- | --- | --- | --- |
| *Pensando alla mia malattia…* | | | | | | | | |
|  |  |  |  |  |  |  |  |  |
|  |  |  |  |  |  |  |  |  |
| *1* | Mi sembra di essere in blackout  O | O | Mi sento in allerta  O | O | Mi sento consapevole  O | O | Mi sento positivo  O |  |
| *2* | Mi sento perduto  O | O | Mi sento in allarme  O | O | Sono cosciente  O | O | Mi sento sereno  O |  |
| *3* | Mi sento sopraffatto dalle emozioni  O | O | Sono in ansia ogni volta che sento un nuovo sintomo  O | O | Sento di essermi abituato alla mia malattia  O | O | Ho un senso di coerenza e continuità nella mia vita nonostante la malattia  O |  |
| *4* | Vivo momenti di grande sconforto  O | O | Mi sento spesso in ansia quando cerco di gestire la mia malattia  O | O | Sento di essermi  adattato alla mia malattia  O | O | Sono tendenzialmente ottimista sul mio futuro e sul mio stato di salute  O |  |
| *5* | Mi sento completamente schiacciato dalla malattia  O | O | Mi agito molto quando appare un nuovo sintomo  O | O | Complessivamente sento di aver accettato la mia malattia  O | O | Riesco a trovare un senso alla mia vita nonostante la malattia  O |  |

**The Patient Health Engagement Scale (PHE-Scale) - English translation for narrative purpose**

Following, you will find 5 statements that describe how a person might feel when thinking about his/her disease. Each sentence can be completed by indicating one of the 4 states or the intermediate points between two states. Please, indicate the state that better describes you by indicating the corresponding position.

| *Thinking about my health status…* | | | | | | | |
| --- | --- | --- | --- | --- | --- | --- | --- |
|  |  |  |  |  |  |  |  |
| *1* | I feel in blackout  O | O | I am in alarm  O | O | I am aware  O | O | I feel positive  O |
| *2* | I feel dazed  O | O | I am in trouble  O | O | I am conscious  O | O | I feel serene  O |
| *3* | When I think about my illness I feel overwhelmed by emotions  O | O | I feel anxious every time a new symptom arises  O | O | I got used to my illness condition  O | O | Despite my illness  I perceive coherence and continuity in my life  O |
| *4* | I feel very discouraged due to my illness  O | O | I feel anxious when I try to manage my illness  O | O | I feel I adjusted to my illness  O | O | I am generally optimist about my future and my health condition  O |
| *5* | I feel totally oppressed by my illness  O | O | I am upset when a new symptom arises  O | O | I feel I have accepted my illness  O | O | I can give sense to my life despite my illness condition  O |
